# Editorial: Advances and challenges in remote monitoring of patients with heart failure

**DOI:** 10.3389/fcvm.2022.1021296

**Published:** 2022-09-12

**Authors:** Leor Perl, Sebastian Feickert, Domenico D'Amario

**Affiliations:** ^1^Cardiovascular Department, Rabin Medical Center, Beilinson Hospital, Petah Tikva, Israel; ^2^The Faculty of Medicine, Tel-Aviv University, Tel Aviv, Israel; ^3^Department of Cardiology, Vivantes Klinikum Am Urban, Berlin and Rostock University Medical Center, Rostock, Germany; ^4^Department of Cardiovascular Sciences, Fondazione Policlinico Universitario Agostino Gemelli Istituto di Ricovero e Cura a Carattere Scientifico (IRCCS), Rome, Italy

**Keywords:** heart failure, monitoring, remote sensing, hemodynamics, fluid overload

Heart failure (HF) is a global pandemic affecting millions worldwide, contributing to high rates of mortality and morbidity, as well as increasing health expenditures, despite significant advances in therapies and prevention and rates are expected to significantly rise in the next years (1, 2).

Remote monitoring of physiological parameters was shown to reduce rates of hospital readmissions for decompensated HF by either invasive hemodynamic sensing systems or structured remote patient management interventions in some trials (3–8), and possibly mortality as well (6, 9, 10). In case of invasive monitoring, it has also proven to be cost-effective in selected patients with HF (11, 12). However, the literature remains equivocal in the evidence of efficacy and safety of these methods. Several trials assessing non-invasive methods to remotely monitor HF patients have failed to show clinical benefit (13–15). Regarding pulmonary artery pressure sensing, the recent GUIDE-HF trial failed to confirm efficacy when it was extended to a wider, real-world setting, including NYHA Class II and IV HF patients (16). A previous clinical trial assessing a right ventricular outlet-based sensing system named Chronicle (Medtronic Inc., Minneapolis, Minnesota) had also failed to reduce the rates of HF-related events (17).

Pressures measured in the right heart do not always adequately correlate with left-sided pressures, leading to inaccurate left-sided filling pressure estimation (18). This is especially true in clinical contexts, such as in patients with non-cardiac-related pulmonary hypertension. A trial assessing left atrial-pressure (LAP) using a sensor lead that is attached to a subcutaneous module, implanted in the atrium through a trans-septal approach was halted prematurely by the safety monitoring board due to a perceived high rate of procedure related complications. However, an analysis made on existing data showed a 41% reduction in HF admissions at 12 months (19, 20).

Monitoring of patients with HF is mostly focused on preventing hospital readmissions. This is reasonable, since HF is the leading cause of hospitalizations among the elderly (21). In fact, the rates of readmissions have even arisen lately, despite readmission reduction programs in the United States (22) and the United Kingdom, especially in lower socio-economic statuses (23). Nevertheless, remote monitoring holds the potential of improving HF patient care by improving the prognosis during admission or enhancing their quality of life in the ambulatory setting.

Chang et al. assessed the ability of models based on machine learning to anticipate the occurrence of cardiogenic shock in a cohort of hospitalized patients who are at increased risk for its development. These models were trained on data spanning 8 years (from 2010 to 2017), from a large regional healthcare system, consisting of 30 hospitals in the United States. The model was designed to predict the need of first cardiogenic shock intervention 2 h ahead, and achieved an overall area under curve (AUC) of 0.87. Interestingly, the authors demonstrated that it can be refined based on specific parameters defining patient subpopulations, such as the presence of HF, which further increases its precision (Chang et al.). Several studies have been published on the value of artificial intelligence in the diagnosis, assessment and prediction of patients with HF (24–28). In the near future, the field of remote monitoring is expected to include these models, as stand-alone or in addition to hardware-based remote-monitoring devices.

One such device is HeartLogic™, a multisensory cardiac implantable electronic device (CIED) based algorithm. It was assessed in the study by Feijen et al. They studied 107 HF patients in a real-world setting, and estimated the accuracy of the system in the prediction of fluid retention, as validated by dedicated HF nurses. For a follow up of 14 months (IQR 8–23), they showed sensitivity of 79%, specificity of 88%, positive predictive value of 71% negative predictive value of 91%. There was a false negative rate of 0.17 alerts/patient year. Importantly, the system could predict which patients required more intense treatment and hospitalization.

Galinier et al. assessed the impact of interventional specialized telemonitoring (ITM), as compared to standard telemonitoring and standard of care in reducing all-cause mortality, cardiovascular mortality and unplanned HF hospitalizations. Four hundred fourteen HF patients from two cohorts in France (OSICAT and ETAPES) were included in this study. The ITM group included patients who were regularly contacted by nurses for therapeutic decisions and guidance. In an event of an alert, cardiologists intervened, adjusted medications and decided on hospitalizations as needed. In the study which lasted a year, there was a lower rate of primary endpoint events in the ITM-group, including all-cause mortality (4.5 vs. 20.2 vs. 16.8%, *p* < 0.05), cardiovascular mortality (3.2 vs. 15.2 vs. 8.4%, *p* < 0.05) and unplanned hospitalizations (13.6 vs. 34.3 vs. 36.8%, *p* < 0.05). This trend remained following multivariable logistic regression (*p* < 0.05 for all endpoints) (Galinier et al.).

Finally, Restivo et al. reported of their 3-year single center experience with the V-LAP™ system, a latest-generation LAP-based device, capable of monitoring pressure wirelessly, *via* an intracardiac lead-less sensor which transmits information to an external device (29). The system is being examined in the V-LAP™ Left Atrium Monitoring system for Patients with Chronic systolic and Diastolic Congestive Heart Failure (VECTOR-HF) trial, which has shown promising initial results (30). In the current study, 5 patients with advanced HF were enrolled, and followed-up for a mean period of 18 months. LAP–based therapy management reduced LAP over time and no hospital readmissions occurred. This result was also accompanied by an improvement in the functional capacity (6-min walking distance 352.5 ± 86.2 meters at baseline to 441.2 ± 125.2 meters at last follow-up) and measures of quality of life (KCCQ overall score 63.82 ± 16.36 vs. 81.92 ± 9.63) (Restivo et al.). This publication joins others showing potential benefit from LAP-based monitoring systems (31–33), but future randomized controlled trials are needed to corroborate this assumption.

In short, the articles published in this Research Topic offer important insights into recent advances in the field of remote monitoring of patients with HF. The rapid development of remote sensing, communication, machine learning, non-invasive methods and the experience gained from clinical trials will surely enable better assessment of patients with HF, improving their wellbeing and reducing their risk of admissions and subsequent adverse events.

## Author contributions

All authors made substantial contribution to the conception or design of the work, or the acquisition, analysis, or interpretation of data for the work and the drafting the work or revising it critically for important intellectual content, provided approval for publication of the content and agree to be accountable for all aspects of the work in ensuring that questions related to the accuracy or integrity of any part of the work are appropriately investigated, and resolved.

## Conflict of interest

Author LP has received consulting fees from Vectorious Medical Technologies and owns stock options in the company. The remaining authors declare that the research was conducted in the absence of any commercial or financial relationships that could be construed as a potential conflict of interest.

## Publisher's note

All claims expressed in this article are solely those of the authors and do not necessarily represent those of their affiliated organizations, or those of the publisher, the editors and the reviewers. Any product that may be evaluated in this article, or claim that may be made by its manufacturer, is not guaranteed or endorsed by the publisher.
